# Identity-by-descent segments in large samples

**DOI:** 10.1016/j.tpb.2025.06.003

**Published:** 2025-07-05

**Authors:** Seth D. Temple, Elizabeth A. Thompson

**Affiliations:** a Department of Statistics, University of Washington, Seattle, WA, USA; b Department of Statistics, University of Michigan, Ann Arbor, MI, USA; c Michigan Institute for Data and AI in Society, University of Michigan, Ann Arbor, MI, USA

**Keywords:** Identity-by-descent, Coalescent, Covariance, Asymptotic normality

## Abstract

If two haplotypes share the same alleles for an extended gene tract, these haplotypes are likely to be derived identical-by-descent from a recent common ancestor. Identity-by-descent segment lengths are correlated via unobserved ancestral tree and recombination processes, which commonly presents challenges to the derivation of theoretical results in population genetics. We show that the proportion of detectable identity-by-descent segments around a locus is normally distributed when the sample size and the scaled population size are large. We generalize this central limit theorem to cover flexible demographic scenarios, multi-way identity-by-descent segments, and multivariate identity-by-descent rates. The regularity conditions on sample size and scaled population size are unlikely to hold in genetic data from real populations, but provide intuition for when the Gaussian distribution may be a reasonable approximate model for the IBD rate. We use efficient simulations to study the distributional behavior of the detectable identity-by-descent rate. One consequence of non-normality in finite samples is that a genome-wide scan looking for excess identity-by-descent rates may be subject to anti-conservative control of family-wise error rates.

## 1. Introduction

Two individuals share a haplotype segment identical-by-descent (IBD) if they inherit it from the same common ancestor. Here, we study the length of IBD segments that overlap a single focal location. Ignoring gene conversion, IBD segments are randomly cut by crossover recombination in each future generation. The length of an IBD segment is thus shorter, with a higher probability, the more removed its common ancestor is from the present day.

Using modern methods, long IBD segments can be detected with high accuracy from genetic data (Freyman et al., 2021; Ramstetter et al., 2017; Shemirani et al., 2021; Zhou et al., 2020b). Detectable segments can provide rich information about the recent genetic history of a population sample. For instance, detected IBD segments have been used to test for rare variant associations when a disease allele is untyped or a genome-wide association study is underpowered (Browning and Thompson, 2012; Gusev et al., 2011; Nait Saada et al., 2020). They have also been used to estimate relatedness (Freyman et al., 2021; Ramstetter et al., 2017; Zhou et al., 2020b), haplotype phase (Browning et al., 2021; Loh et al., 2016), mutation rates (Palamara et al., 2015; Tian et al., 2019, 2022), recombination rates (Zhou et al., 2020c), gene conversion rates (Browning and Browning, 2024; Palamara et al., 2015), demographic changes (Browning and Browning, 2015; Cai et al., 2023; Palamara et al., 2012), and positive selection (Temple et al., 2024). We will study the sample mean of indicators if an IBD segment is long enough to be reliably detected. The binary random variables are correlated via unobserved recombinations and a random ancestral tree. IBD segments longer than 0.02 Morgans (defined below) can be detected with high accuracy in high-quality genetic data (Temple et al., 2025).

For independent, identically distributed data, maximum likelihood estimators are asymptotically consistent, efficient, and normally distributed under regularity conditions (Section 10.6.2 in Casella and Berger (2002)). Composite likelihood approaches are commonly used in genetics when it is analytically intractable or computationally expensive to address dependencies in the data (Larribe and Fearnhead, 2011). To what extent consistency, efficiency, and asymptotic normality extend to maximum composite likelihood estimators is generally unknown (Larribe and Fearnhead, 2011). Studying maximum composite likelihood estimators can be especially challenging if their maxima do not have a closed form (Palamara et al., 2012; Tian et al., 2019). In our work, the composite likelihood will be the binomial likelihood, which is maximized by the sample mean of binary random variables. The statistical property we care about the most is asymptotic normality, which means that the estimator’s distribution converges to a Gaussian distribution as the sample size tends to infinity (Casella and Berger, 2002).

Without theoretical results, some authors assume that their estimators are distributed within some parametric family. In one example, Palamara et al. (2018) assume without proof that their estimator of coalescent rates within the past tens of generations is Gamma distributed. In another example, Carmi et al. (2013) observe that the Gaussian distribution is a good fit for the average fraction of the genome shared IBD by an individual with any other individual in a population sample. Still, this empirical observation is not the same as a theoretical result. When the sampling distribution is not subnormal (Wainwright, 2019), statistical inference assuming normality may understate the probability of extreme values.

Creating valid confidence intervals can be more straightforward when an estimator is asymptotically normally distributed. The parametric bootstrap approach proposed in Temple et al. (2024) gives adequate coverage in selection coefficient estimation for numerous simulation studies. Their technique implicitly assumes that the rate of detectable IBD segments around a locus, and certain functions thereof (Theorem 5.5.24 in Casella and Berger (2002)), are normally distributed in large samples. In contrast, bootstrap resampling (Efron, 1987) has been employed in IBD-based estimation procedures (Browning and Browning, 2015, 2024; Cai et al., 2023; Palamara et al., 2012; Tian et al., 2019). For significance level α, these existing works do not demonstrate that their (1-α)% bootstrap confidence intervals contain a true parameter in (1-α)% of simulations. Moreover, nonparametric bootstrapping tends to give confidence intervals that are not wide enough to satisfy coverage (Manly, 2018).

Here, we derive sufficient conditions under which the proportion of detectable IBD segments around a locus is asymptotically normally distributed. The proof is to show that the variance of detectable IBD segments dominates the covariance between detectable IBD segments. Our conditions involve a minimum length of detectable IBD segments, multiplied by the population size from which a large sample is drawn. The large population size requirement, in particular, indicates that most of the branch lengths in the ancestral tree must be long for the result to hold. The overall contribution of this work is to support IBD-based statistical inference with rigorous theory and extensive simulation studies.

The outline of the paper is as follows. In Section 2, we formally define our probability model for IBD segments that overlap a fixed location. In Section 3, we present and prove our main result for the asymptotic normality of the detectable IBD rate around a fixed location. In Section 4, we generalize our central limit theorem to cover nonconstant population sizes, multi-way IBD segments, and IBD rates between samples from the same population. In Section 5, we use simulation to investigate the statistical properties of IBD-based estimators and IBD graphs around a locus. Many calculations of covariance terms are left to Appendix A.1.

## 2. Preliminary material

First, we define our mathematical notation (Table 1). The notation in Sections 2.1 and 2.2 follows the notation used in Temple et al. (2025). We use the Kingman coalescent (Kingman, 1982b,a) as a model for the times until recent common ancestors. We model recombination using the classical model of Haldane (1919) under which crossovers occur as a Poisson process. The probability that an IBD segment is longer than a detection threshold is derived by integrating over these two waiting time distributions.

### 2.1. The time until a common ancestor

Let n be the haploid sample size and k≤n be the size of a subsample. Define N to be the constant population size. Let the random variable $T_{k}$ denote the time until a common ancestor is reached for any two of k haploids, which we measure in units of N generations. In the discrete-time Wright-Fisher (WF) process, each haploid individual has a haploid ancestor in the previous generation, and if haploids share the same haploid ancestor, their lineages merge.

The Kingman coalescent comes from a continuous-time scaling limit of the WF process when subsample sizes are much smaller than constant population sizes. Specifically, $T_{k}$ converges weakly to Exponential k2 for k≪N and N→∞ (Kingman, 1982b,a), where k2 is the rate parameter. For the proofs of our theoretical results, we consider the marginal covariances of IBD segments involving two, three, and four specific haplotypes. As a result, we focus only on the times $T_{4}∼\text{Exponential}(6)$, $T_{3}∼\text{Exponential}(3)$, and $T_{2}∼\text{Exponential}(1)$ until any two of the four, three, and two haploids reach a common ancestor, respectively.

### 2.2. The distance until crossover recombination

The genetic distance (in Morgans) between two loci is the number of crossovers expected to occur in an offspring gamete. Assuming independent crossovers, Haldane (1919) derives that the genetic distance until a crossover recombination is exponentially distributed. This result leads to modeling crossover points along the genome as a Poisson process. Browning (2000) considers some other crossover models (Kosambi, 1943) when studying transitions between IBD states, whereas we exclusively use the Haldane model.

From a fixed point, the Morgans distance in one direction until a gamete offspring crossover is exponentially distributed with rate parameter 1. After t independent meioses, the surviving haplotype segment length to the right of the focal location is distributed as Exponential(t), where t is the rate parameter. Note that our model concerns recombinations around a focal point, whereas the sequentially Markovian coalescent concerns recombinations along the genome (Hein et al., 2004; Li and Durbin, 2011; Schiffels and Durbin, 2014). Let a and b be sample haplotypes in the current generation, and define $L_{a},R_{a}|t∼\text{Exponential}(t)$ to be sample haplotype a’s recombination endpoints to the left and right of a focal location after t generations. Because crossovers to the left and right of the focal location are independent, the extant width from the ancestor at time t is $W_{a}:=L_{a}+R_{a}|t∼Gamma(2,t)$. Since the t meioses descend independently to a and b from their most recent common ancestor, the IBD segments that are shared by a and b are $L_{a,b},R_{a,b}|t∼Exponential(2t)$ and $W_{a,b}:=L_{a,b}+R_{a,b}|t∼Gamma(2,\;2t)$.

### 2.3. The presence of detectable IBD segments

Relative to a focal point, we consider the detection of long IBD segments in a sample. Let $X_{a,b}:=X_{a,b}(w)=I\left(R_{a,b}\geq w\right)$ indicate if the IBD segment to the right that is shared by sample haplotypes a and b is longer than a detection threshold w Morgans. The binary random variables $\left\{X_{a,b}\right\}$ are identically distributed with the same mean $E_{2}\left[X_{a,b}\right]$ and correlated through the unobserved coalescent tree. We use $E_{2},\;E_{3}$, and $E_{4}$ and ${\text{Cov}}_{2}$, ${\text{Cov}}_{3}$, and ${\text{Cov}}_{4}$ to denote expected values and covariances with respect to coalescent trees of two, three, and four sample haplotypes, respectively.

Our central limit theorem concerns the mean of the IBD segment indicator random variables. Namely, the detectable IBD rate to the right of a fixed location is

(1) X‾n2:=n2-1∑(a,b)Xa,b.

Let $Z_{a,b}:=X_{a,b}-E_{2}\left[X_{a,b}\right]$ be the mean-centered binary random variable, and let the sum of all except one of these mean-centered random variables be $Z_{-a,b}:=\sum_{\left(c,d\right)}Z_{c,d}-Z_{a,b}$. The sum of variances of all IBD segment indicators is

(2) Ωn2:=∑(a,b)VarXa,b=n2×E2Xa,b×1-E2Xa,b.

Finally, the mean-centered and suitably scaled detectable IBD rate to the right of a locus is

(3) Z‾n2:=Ωn2-1/2×(∑a,bXa,b-E2Xa,b).

We use the subscript nk to denote when the mean is over nk combinations of k haplotypes.

**Fig. 1.**
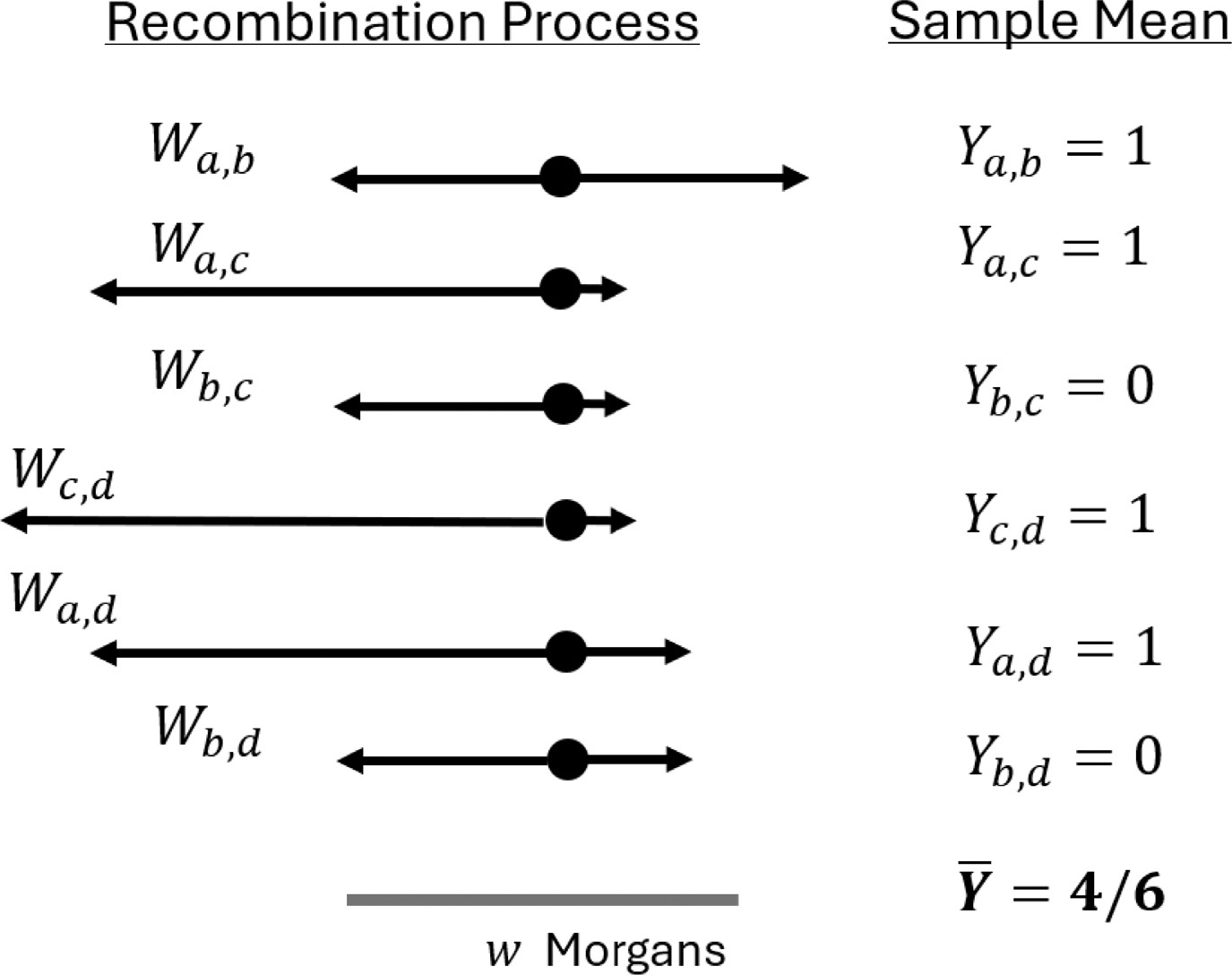
Example calculation of the detectable IBD rate. IBD segment lengths overlapping a focal point for sample haplotypes a,b,c,d are shown. The IBD segment indicators ($Y_{i,j}$’s) are 1 if their IBD segment lengths ($W_{i,j}$’s) exceed w Morgans and otherwise 0. The detectable IBD rate $\bar{Y}$ is the mean of these correlated binary random variables. The detectable IBD rate to the right of the focal point, $\bar{X}$, is calculated similarly.

For IBD segments overlapping a focal location, let $Y_{a,b}:=I\left(L_{a,b}+\right.\left.R_{a,b}\geq w\right)$ and ${\tilde{Z}}_{a,b}:=Y_{a,b}-E_{2}\left[Y_{a,b}\right]$. The terms Y‾n2,Z˜-a,b,Z˜¯n2, and Ω˜n2, are defined analogously to X‾n2,Z-a,b,Z‾n2, and Ωn2, respectively. We drop the subscript n2 when it is clear that the aggregation is over n2 pairs of haplotypes. Fig. 1 provides a conceptual example calculating $\bar{Y}$ for four sample haplotypes.

We use additional subscript indices when segments are IBD among multiple haplotypes, which we refer to as multi-way IBD segments. For instance, $Y_{a,b,c}$ indicates whether the IBD segment around a locus shared between haplotypes a,b, and c is longer than w Morgans. The corresponding sample mean over n3 haplotype triplets is denoted Y‾n3, and the related sums, means, and variances are defined similarly. This notation is important to extend our main central limit theorem to multi-way IBD segment indicators.

We use the superscript l to denote the sample label when different population samples are considered. For example, $X_{a,b}^{0}$ and $X_{c,d}^{1}$ indicate if the IBD segments to the right of a locus that are shared between haplotypes a and b in population sample 0 and c and d in population sample 1 are longer than w Morgans, respectively. IBD segments around a locus and mean-centered terms are defined analogously for these extensions. For example, the mean in population sample 0 of 2-way IBD segment indicators overlapping a focal location is denoted ${\bar{Y}}^{0}$. This notation is important to extend our main univariate central limit theorem to a multivariate Gaussian version.

## 3. Main central limit theorem

If $U_{1},\ldots ,U_{n}{∼}^{\mathrm{iid}}G$ for some model G, the Lindeberg–Lévy central limit theorem says that the standardized sample mean weakly converges to the standard normal distribution (under some regularity conditions) (Lindeberg, 1922). The special case of this result for binary random variables (De Moivre, 1718) is more closely related to our work. The result does not apply in our case because the IBD segment indicators $\left\{X_{a,b}\right\}$ to the right of a focal point are not independent.

We start by focusing on the mean-centered and suitably scaled detectable IBD rate Z‾n2,N to the right of a focal location, where the subscript N clarifies that the haplotypes are sampled from a population of constant size N. Our central limit theorems concern large sample size n and large population size N scaled by the Morgans detection threshold w. The intuition for our weak law is that the covariance between IBD segment indicators $\sum_{\left(a,b)\neq (c,d\right)}Cov\left(X_{a,b},X_{c,d}\right)$ is small relative to the sum of the variances of the individual IBD segment indicators Ωn2.

We will use the result referred to as Corollary 2 of Theorem 4 in Chandrasekhar and Jackson (2025) and Corollary 1 of Theorem 1 in Chandrasekhar et al. (2023) in our proof. The sum of covariances between random variables being negligible compared to the sum of variances of the random variables themselves is the basis of the general central limit theorem for dependent data that is given in Chandrasekhar and Jackson (2025) and Chandrasekhar et al. (2023). For univariate identically distributed binary random variables $X_{1},\ldots ,X_{n}$, the main condition in Chandrasekhar et al. (2023) and Chandrasekhar and Jackson (2025) to satisfy is that $\sum_{i=1}^{n}Var\left(X_{i}\right)$ is of the same little “o” order as $\sum_{i\neq j}Cov\left(X_{i},X_{j}\right)$.

### Theorem 3.1.

*For* n *and* Nw *tending to infinity, the mean-centered and suitably scaled detectable IBD rate* Z‾n2,N *to the right of a focal location converges in distribution to the standard normal distribution when the following are true:*

1. $Nw=o\left(n^{2}\right)$, *scaled population size is small relative to the number of pairs;*
2. n=o(Nw), sample size is small relative to scaled population size;
3. $E\left[Z_{a,b}\times Z_{-a,b}|Z_{-a,b}\right]\geq 0$ *for every* $Z_{a,b}$.

### Proof.

**Fig. 2.**
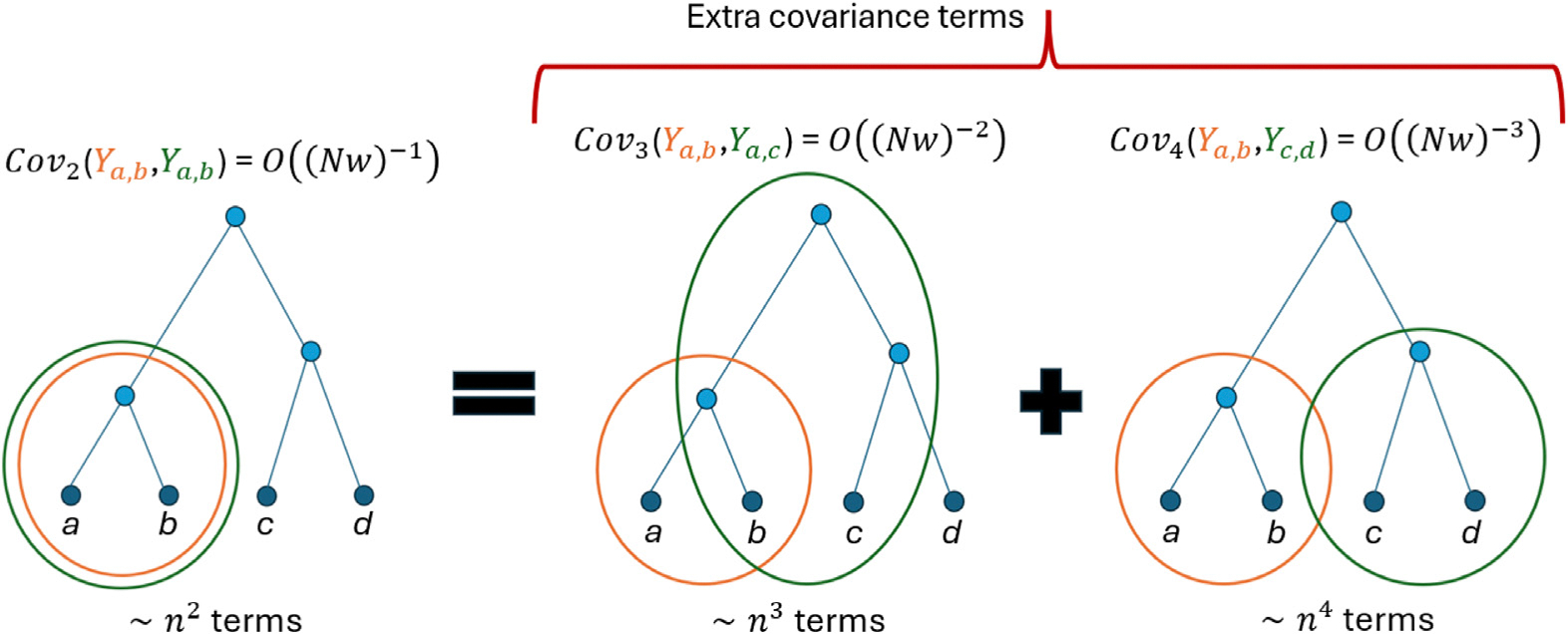
Schematic diagram for the theorem proofs. The strategy is to show that the additional terms in the total covariance (to the right of the equals sign) are of the same little o(⋅) order as if the identically distributed $\left\{Y_{a,b}\right\}$ were independent. The coalescent tree of haplotypes a,b,c,d is shown. The covariances ${Cov}_{2}\left(Y_{a,b},Y_{a,b}\right),\;{Cov}_{3}\left(Y_{a,b},Y_{a,b}\right)$, and ${Cov}_{4}\left(Y_{a,b},Y_{c,d}\right)$ for IBD segments overlapping the focal point are calculated by integrating over haplotype segment lengths and the branches of the tree contained by the orange and green circles. Upper bounds on the covariances for IBD segments to the right of the focal point are derived in Eq. (5) and Lemmas A.3 and A.4, and upper bounds on the covariances for IBD segments overlapping the focal point are derived in Lemma A.5. The asymptotically equivalent numbers of the marginal covariances ${Cov}_{2}\left(Y_{a,b},Y_{a,b}\right),\;{Cov}_{3}\left(Y_{a,b},Y_{a,b}\right)$, and ${Cov}_{4}\left(Y_{a,b},Y_{c,d}\right)$ are given below the trees.

We show that our three conditions are sufficient to apply Corollary 1 in Chandrasekhar et al. (2023). Fig. 2 depicts the general strategy we take to prove this theorem and our subsequent theorems. Without loss of generality, we derive integrals over a tree with two sample haplotypes a and b, a tree with three sample haplotypes a,b, and c, and a tree with four sample haplotypes a,b,c, and d. In all our expected value calculations, we start by having already integrated over the recombination endpoints, which gives the survival function at the detection threshold w for Gamma shape parameters 1 or 2. For example, the first line of

(4) $$E_{2}\left[X_{a,b}\right]=\int \;S^{2}\left(w;1,Nw\right)\cdot h\left(t_{2}\right)dt_{2},$$

where S(w;α,Nw) is the survival function of a Gamma random variable with shape parameter α=1 and rate parameter $Nw,h\left(t_{2}\right)$ is the exponential density with rate parameter $t_{2}$, and there are two independent recombination endpoints greater than w. This calculation simplifies to

(5) $$E_{2}\left[X_{a,b}\right]\;=\int exp\left(-2Nt_{2}w\right)\;exp\left(-t_{2}\right)\;dt_{2}\;\;=(2Nw+1{)}^{-1}\int \;(2Nw+1)exp\left(-(2Nw+1)t_{2}\right)\;dt_{2}\;\;=(2Nw+1{)}^{-1}=O((Nw{)}^{-1}).$$

One technique for calculating the integrals in this paper is to rearrange the integrand in the form of exponential densities. It is easy to show that $E_{2}\left[X_{a,b}\right]\to 0$ uniformly for large scaled population size (Lemma A.1). The condition $Nw=o\left(n^{2}\right)$ implies that Ωn2→∞ (Eqs. (2) and (5)). The assumption in Chandrasekhar et al. (2023) that $E[{\left|Z_{a,b}\right|}^{3}]/E{\left[{\left|Z_{a,b}\right|}^{2}\right]}^{3/2}$ is bounded above is true for nondegenerate Bernoulli random variables (Chandrasekhar and Jackson, 2025) (Lemma A.2). Lastly, given n=o(Nw), we show that

(6) ∑(a,b)≠(c,d)CovXa,b,Xc,d=∑a,b,cCov3Xa,b,Xa,c+∑a,b,c,dCov4Xa,b,Xc,d=o(Ωn2).

In Appendix A.1, we derive bounds on the integrals ${Cov}_{3}\left(X_{a,b},X_{a,c}\right)=O((Nw{)}^{-2})$ and ${Cov}_{4}\left(X_{a,b},X_{c,d}\right)=O((Nw{)}^{-3})$. Next, there are $n(n-1)(n-2)∼n^{3}$ combinations of three haplotypes a,b, and c, and there are $n(n-1)(n-2)(n-3)/4∼n^{4}$ combinations of four haplotypes a,b,c, and d. In asymptotic arguments, the notation ∼ means asymptotic equivalence, not distributed as.


(7) Ω(n2)∼n2⋅O((Nw)-1)=o((Nw)2)⋅O((Nw)-1)=o(Nw);

(8) $$\sum_{a,b,c}{Cov}_{3}(X_{a,b},X_{a,c})∼n^{3}\cdot O((Nw{)}^{-2})=o((Nw{)}^{3})\cdot O((Nw{)}^{-2})=o\left(Nw\right);$$

(9) $$\sum_{a,b,c,d}{Cov}_{4}\left(X_{a,b},X_{c,d}\right)∼n^{4}\cdot O((Nw{)}^{-3})=o((Nw{)}^{4})\cdot O((Nw{)}^{-3})=o(Nw).$$

The covariance between IBD segment indicators (Eqs. (8) and (9)) is controlled by the covariance within IBD segment indicators (Eq. (7)). □

The first two conditions have appealing interpretations. First, when $Nw=o\left(n^{2}\right)$, the sample size squared is large enough relative to the scaled population size such that we expect to observe many IBD segments to the right of a focal location that are longer than the Morgans threshold w (Eq. (5)). Second, as Nw increases the marginal covariance terms ${Cov}_{3}\left(X_{a,b},X_{a,c}\right)$ and ${Cov}_{4}\left(X_{a,b},X_{c,d}\right)$ shrink much faster than ${Cov}_{2}\left(X_{a,b},X_{a,b}\right)$. Thus, when n=o(Nw), we do not observe many large clusters of haplotypes with IBD segments to the right of a focal location that are longer than the Morgans threshold w so long as the sample size is not too large relative to the scaled population size.

These two conditions are unlikely to hold in genetic data from real populations; however, they provide intuition for when the Gaussian distribution may be a reasonable approximate model for the IBD rate. Figure S1 shows the limiting behavior of $Nw/n^{2}$ and n/(Nw) as sample size n and population size N increase. For humans, we have genetic data from 10s to 100s of thousands of humans who come from populations with recent effective sizes on the order of $10^{7}$, in which case these values $Nw/n^{2}$ and n/(Nw) with 0.01≤w≤0.04 are small but far from zero.

To consider rates of convergence, we fix $Nw=C_{1}\cdot n^{C_{2}}$, where $C_{1}>0$ and $1<C_{2}<2$. In this case, the following proposition implies that the best possible rate of convergence is $n^{1/2}$. This fastest rate matches the convergence rate of the Lindeberg–Lévy central limit theorem.

### Proposition 3.2.

The rate of convergence is $min(n^{-\left(1-C_{2}\right)},n^{2-C_{2}})$. Therefore, the fastest rate of convergence is $n^{1/2}$ when $C_{2}=3/2$.

#### Proof.

The conditions $Nw=o\left(n^{2}\right)$ and n=o(Nw) in Theorem 3.1 mean that the following two limits approach 0.


(10) $$\underset{n\to \infty }{lim}Nw/n^{2}=\underset{n\to \infty }{lim}C_{1}\cdot n^{C_{2}-2}=0.$$

(11) $$\underset{n\to \infty }{lim}n/(Nw)=\underset{n\to \infty }{lim}C_{1}^{-1}\cdot n^{1-C_{2}}=0.$$

The rate of convergence is the smaller of the two convergence rates in these limits. □

The third condition also has an interpretation in the context of population genetics. Figure S2 provides a diagram that builds intuition for this condition. The statement says that if the number of detectable IBD segments to the right of a focal location, except for $X_{a,b}$, is less than the expectation EXa,b×n2-1, then the IBD segment to the right of a focal location that is shared by a and b is shorter than w Morgans on average, and vice versa if $X_{-a,b}$ is greater than its expected value. This assumption seems plausible if IBD segments to the right of a focal location have nonnegative covariance (when Nw is large), which we verify from the expected value Equations A.3, A.4, A.5, A.7, A.8, A.10, and A.11 in the proofs of Lemmas A.3 and A.4. Moreover, one intuits that the unobserved coalescent tree has longer branch lengths when we observe fewer IBD segments than expected. That is, the posterior distribution of $X_{a,b}|X_{-a,b}$ is more likely to come from a tree with long branches than the unconditional distribution of $X_{a,b}$ is when X-a,b<E2Xa,b×n2-1, and vice versa when X-a,b>E2Xa,b×n2-1.

One can show that the small sample size n=3 is a pathological example where the third condition breaks down (Lemma A.6). We do not otherwise calculate $E\left[Z_{a,b}\times Z_{-a,b}|Z_{-a,b}\right]$ for all $Z_{-a,b}$, which involves integration over the space of all coalescent trees and the 2n2-1 hypercube of 0 ‘s and 1 ‘s. In a simulation study, we evaluate the third condition via the Monte Carlo method (Appendix A.2), concluding that this condition likely holds in large samples.

The asymptotic normality of Z˜¯n2,N follows from the same arguments as those of the proof in Theorem 3.1. We show in Appendix A. 1 that ${Cov}_{2}\left(Y_{a,b},Y_{a,b}\right),{Cov}_{3}\left(Y_{a,b},Y_{a,c}\right)$, and ${Cov}_{4}\left(Y_{a,b},Y_{c,d}\right)$ are $O((Nw{)}^{-1}),\;O((Nw{)}^{-2})$, and $O((Nw{)}^{-3})$, respectively.

### Theorem 3.3.

*For* n *and* Nw *tending to infinity, the mean-centered and suitably scaled detectable IBD rate* Z˜¯n2,N *around a locus converges in distribution to the standard normal distribution when the following are true:*

1. $Nw=o\left(n^{2}\right)$
2. n=o(Nw)
3. $E\left[{\tilde{Z}}_{a,b}\times {\tilde{Z}}_{-a,b}|{\tilde{Z}}_{-a,b}\right]\geq 0$ *for every* ${\tilde{Z}}_{a,b}$.

All our proofs involve calculating the covariances between detectable IBD segments around a focal point. Carmi et al. (2013) also derive (approximate) covariance formulas for a particular sample mean that depends on IBD segments longer than a detection threshold, except they consider IBD segments along the entire genome (Equation 27 in Carmi et al. (2013)). In Appendix A.3, we draw connections between our covariance formulas and a covariance formula in Carmi et al. (2013).

## 4. Extensions

### 4.1. Flexible demographic scenarios

We can derive a similar result for varying population sizes. Let $N_{1}={max}_{t}N(t)$ and $N_{2}={min}_{t}N(t)$. Compared to varying population sizes N(t), the indicator of a detectable IBD segment around a focal location has a larger expected value when sample haplotypes come from a constant population of size $N_{2}$. Conversely, compared to varying population sizes N(t), the indicator of a detectable IBD segment around a focal location has a smaller expected value when sample haplotypes come from a constant population of size $N_{1}$. We use these facts to establish covariance bounds for complex demography.

#### Theorem 4.1.

*For* $n,\;N_{1}w$, *and* $N_{2}w$ *tending to infinity, the mean-centered and suitably scaled detectable IBD rate* Z‾n2,N(t) *to the right of a focal location converges in distribution to the standard normal distribution when the following are true:*

1. $N_{1}w=o\left(n^{2}\right);$
2. $n=o\left(N_{2}w\right);$
3. $E\left[Z_{a,b}\times Z_{-a,b}|Z_{-a,b}\right]\geq 0$ *for every* $Z_{a,b}$.

*The same conditions imply weak convergence for* Z˜¯n2,N(t).

#### Proof.

The argument is the same as in Theorem 3.1, except we use $N_{1}$ and $N_{2}$ to upper and lower bound covariance terms.


(12) Ωn2∼n2⋅O((N2w)-1)=oN2w;

(13) $$\sum_{a,b,c}{Cov}_{3}(X_{a,b},X_{a,c})∼n^{3}\cdot O({\left(N_{2}w\right)}^{-2})=o\left(N_{2}w\right);$$

(14) $$\sum_{a,b,c,d}{Cov}_{4}(X_{a,b},X_{c,d})∼n^{4}\cdot O({\left(N_{2}w\right)}^{-3})=o\left(N_{2}w\right).\;□$$

Theorem 3.1 is a special case of Theorem 4.1 when $N_{1}=N_{2}$. The conditions in Theorem 4.1 are unlikely to hold in real data examples and are more challenging to interpret. Note that the proof of Theorem 4.1 does not make use of the entire curve N(t). The population sizes at the most recent coalescent times have the greatest impact on the covariance of and between IBD segments around a focal location. As in Theorem 3.3, we can extend Theorem 4.1 to address IBD segments overlapping a focal location.

### 4.2. Multi-way IBD segments

To calculate the probability that an m-way IBD segment indicator is 1, we integrate over m-1 coalescent times and the recombination processes at these common ancestors. Here, we consider m>2 but m much smaller than the sample size n. For example, we compute the expected value of the 3-way IBD segment indicator to the right of a focal location.


(15) $$E_{3}\left[X_{a,b,c}\right]\;=\int exp\left(-2Nt_{2}w\right)\;exp\left(-3Nt_{3}w\right)\;exp\left(-t_{2}\right)\;exp\left(-3t_{3}\right)\;dt_{2}dt_{3}\;\;=3(2Nw+1{)}^{-1}(Nw+1{)}^{-1}=O((Nw{)}^{-2})$$

Note this derivation and that of Eq. (5) fall under the general result that $E_{m}\left[X_{\ldots m}\right]=O((Nw{)}^{-(m-1)})$, where …m denotes m labeled haplotypes. To observe many m-way IBD segment indicators, we require $(Nw{)}^{m-1}=o\left(n^{m}\right)$ because the sums are over nm∼nm identically distributed random variables.

#### Theorem 4.2.

*For* n *and* Nw *tending to infinity and bounded* m=O(1), *the mean-centered and suitably scaled detectable IBD rate* Z‾nm,N *to the right of a focal location converges in distribution to the standard normal distribution when the following are true:*

1. $(Nw{)}^{m-1}=o\left(n^{m}\right);$
2. n=o(Nw);
3. $E\left[Z_{\ldots m}\times Z_{-\ldots m}|Z_{-\ldots m}\right]\geq 0$ *for every* $Z_{\ldots m}.$

The weak convergence result holds for Z˜¯nm,N under the same conditions.

#### Proof.

The proof is again to show that the three conditions are sufficient to apply Corollary 1 in Chandrasekhar et al. (2023). The strategy is to calculate the relevant integrals $E_{m}[\cdot ],\ldots ,E_{2m}[\cdot ]$, count the number of occurrences of each covariance type, and then observe that the condition n=o(Nw) is sufficient to control the total covariance. In Appendix A.1.2, we give a full proof for the 3-way IBD rate, from whose covariances and combinatorics it is straightforward to see a pattern as m increases. □

Theorems 3.1 and 3.3 are special cases of Theorem 4.2 when m=2. We remark that n=o(Nw), which does not involve m, is a condition shared between Theorems 3.1 and 4.2. Recall that this condition maintains that covariances between IBD segment indicators are small, which is governed by large scaled population size Nw.

### 4.3. Multi-sample IBD rates

We now show that the conditions n=o(Nw) and $Nw=o\left(n^{2}\right)$ are also sufficient to apply the multivariate version of the Chandrasekhar et al. (2023) central limit theorem. From the multivariate result, we can derive the asymptotic distribution of the difference in IBD rates between the disjoint sample sets. This test statistic may be useful in examining the IBD rates of case individuals with a disease-related trait versus control individuals without the disease-related trait.

To extend our main result to the IBD rates of different samples from a population, we consider the example of two disjoint sample sets labeled 0 and 1. Each sample consists of n samples from the same population of size N. Let ${\left({\bar{X}}^{0},{\bar{X}}^{1}\right)}^{\prime }\in R^{2}$ be the vector of two sample means, where ′ is transpose. The detectable identity-by-descent segment rates around a locus are denoted ${\left({\bar{Y}}^{0},{\bar{Y}}^{1}\right)}^{\prime }$, and the standardized sample means are denoted ${\left({\bar{Z}}^{0},{\bar{Z}}^{1}\right)}^{\prime }$ and ${\left({\bar{\tilde{Z}}}^{0},{\bar{\tilde{Z}}}^{1}\right)}^{\prime }$. In general, we denote ${\bar{X}}^{1:\ell }:={\left({\bar{X}}^{0},{\bar{X}}^{1},\ldots ,{\bar{X}}^{\ell -1}\right)}^{\prime }$ and ${\bar{Y}}^{1:\ell }:={\left({\bar{Y}}^{0},{\bar{Y}}^{1},\ldots ,{\bar{Y}}^{\ell -1}\right)}^{\prime }$ IBD rates to the right of and overlapping a focal location for ℓ distinct samples of n haplotypes from N. The (element-wise) standardized versions of these IBD rates are ${\bar{Z}}^{1:\ell }$ and ${\bar{\tilde{Z}}}^{1:\ell }$, respectively. For the l th sample, the mean-centered sums of IBD segment indicators excluding $Z_{a,b}^{l}$ and ${\tilde{Z}}_{a,b}^{l}$ are denoted $Z_{-a,b}^{l}{\tilde{Z}}_{-a,b}^{l}$, respectively.

#### Theorem 4.3.

*For* n *and* Nw *tending to infinity and finite* ℓ, *the mean-centered and suitably scaled IBD rates* ${\bar{Z}}^{1:\ell }$ *converge in distribution to the standard normal distribution* $N_{\ell }\left(0,I_{\ell \times \ell }\right)$ *when the following are true:*

1. $Nw=o\left(n^{2}\right);$
2. n=o(Nw);
3. $E\left[Z_{a,b}^{l}\times Z_{-a,b}^{l}|Z_{-a,b}^{l}\right]\geq 0$ *for every* $Z_{a,b}^{l}.$

*The weak convergence result holds for* ${\bar{\tilde{Z}}}^{1:\ell }$ *under the same conditions. (The proof is in* Appendix A.1.3 *using the result from* Chandrasekhar et al. (2023).)

One important consequence of Theorem 4.3 is that affine transformations of the sample means column vector are asymptotically normally distributed. In particular, for the example of two samples and the row vector (1, −1), the difference in standardized IBD rates around a locus ${\bar{\tilde{Z}}}^{1}-{\bar{\tilde{Z}}}^{0}$ is asymptotically normally distributed. When there are ℓ sample sets, for each pair of the ℓ sample means, a row vector exists such that the dot product gives the difference in their IBD rates.

To apply Corollary 1 of Chandrasekhar et al. (2023), we restrict our result to equally sized samples of n haplotypes. In case-control studies, there may be samples of unequal sizes $n_{1}$ and $n_{0}$. We conjecture that the difference in IBD rates will still be asymptotically normally distributed, so long as $Nw=o\left(n_{1}^{2}\right)$ and $Nw=o\left(n_{0}^{2}\right)$ and $max\left(n_{0},n_{1}\right)=o(Nw)$. The conditions $Nw=o\left(n_{1}^{2}\right)$ and $Nw=o\left(n_{0}^{2}\right)$ maintain that we detect many IBD segments in both samples. The condition $max(n_{0},n_{1})=o(Nw)$ maintains that covariances are vanishing both in the diagonal terms $Cov({\bar{\tilde{Z}}}^{1},{\bar{\tilde{Z}}}^{1})$ and $Cov({\bar{\tilde{Z}}}^{0},{\bar{\tilde{Z}}}^{0})$ and the off-diagonal term $Cov({\bar{\tilde{Z}}}^{0},{\bar{\tilde{Z}}}^{1})$. Another limitation is our restriction to distinct sample sets, which is necessary to make the covariance calculations analytically tractable.

## 5. Simulation studies

The theoretical results in Sections 3 and 4 rely on asymptotic conditions, not finite sample conditions. Using simulation, we explore the finite sample empirical distributions and percentiles of detectable IBD rate-based statistics around a fixed location. To investigate normality, we require massive simulations to form tens of thousands of empirical distributions.

**Fig. 3.**
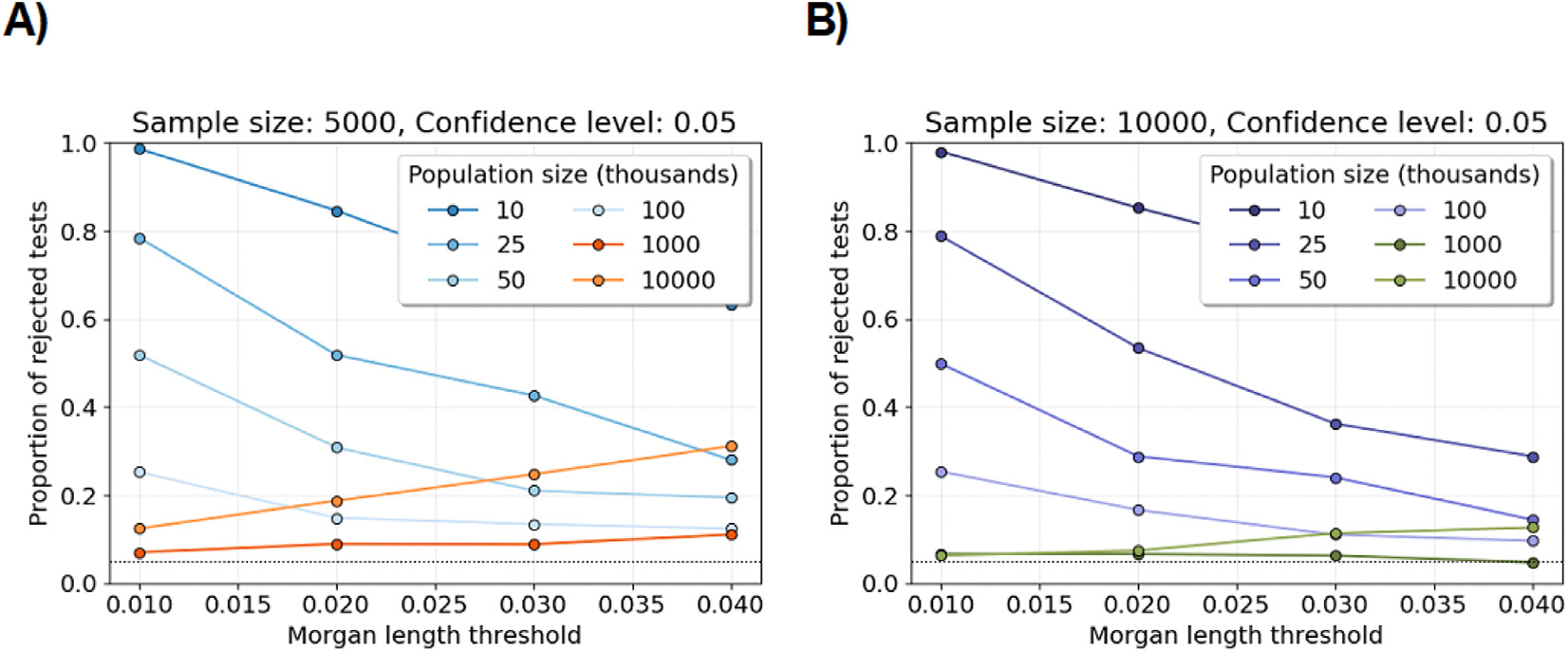
Shapiro–Wilk tests for varying population sizes. Line plots show the proportions of Shapiro–Wilk tests rejected at the significance level of 0.05 (y-axis) for varying population sizes and a fixed sample size. Each proportion is computed over five hundred tests. Each test is based on 1000 simulations of the number of identity-by-descent lengths longer than a specified Morgans length threshold (x-axis). (A) The sample size consists of 5000 individuals. (B) The sample size is 10,000. The legends assign colors to different population sizes. The horizontal dotted line is at 0.05.

We use the algorithm in Temple et al. (2025) to simulate detectable IBD segments overlapping a fixed location. The algorithm first simulates coalescent times, then simulates recombination endpoints to the left and right of the focal point, and finally makes as few computations as possible to derive the IBD segments longer than the detection threshold. The algorithm makes significantly fewer computations than n2 by ignoring haplotype pairs once either of their segment lengths is smaller than the detection threshold. Temple et al. (2025) show that their method simulates an IBD segment length distribution similar to existing methods (Palamara, 2016; Guo et al., 2024) (Figures S7, S8, and S10 in Temple et al. (2025)). They also demonstrate that their method’s runtime scales approximately linearly with the sample size, whereas the runtimes of existing methods (Palamara, 2016; Guo et al., 2024) scale quadratically (Figs. 2, 3, and S6, and Table 2 in Temple et al. (2025)).

Despite the speed of the algorithm in simulating as many as n2 IBD segment lengths, the enormous scope of our simulations takes hundreds of days of computing time, which we spread across core processing units. If not for the algorithm’s efficiency, we would be limited in our ability to study the distributional behavior of the standardized detectable IBD rate $\bar{\tilde{Z}}$ and the difference in IBD rates ${\bar{\tilde{Z}}}^{1}-{\bar{\tilde{Z}}}^{0}$.

We consider sample sizes of 5000 and 10,000 “diploid” individuals. To implement “diploids”, we use a haploid model with two times the sample size of diploids (and likewise for demographic models). We consider the same demographic scenarios described in Temple et al. (2024) and Temple et al. (2025): constant population sizes ranging from 10,000 to 10 million diploid individuals, as well as examples of exponential growth phases and a population bottleneck. Both complex demographic scenarios amount to population sizes ≥10^6^ in the most recent tens of generations and population sizes ≤10^4^ more than a few hundred generations ago. Figure S3 from Temple et al. (2025) illustrates some of these demographic scenarios.

### 5.1. Identity-by-descent rates in finite samples

#### 5.1.1. Constant population sizes

Using the Shapiro–Wilk test (Shapiro and Francia, 1972; Shapiro and Wilk, 1965; Shapiro et al., 1968), we investigate if empirical distributions of $\sum_{a,b}Y_{a,b}$ resemble normal distributions as sample size n, population size N, and the Morgans length threshold w increase. We partition simulated IBD rates into 500 empirical distributions, where each empirical distribution is based on 1000 observations. The null hypothesis is that the empirical distribution of detectable IBD rates is normally distributed. We report the proportion of times we reject the null hypothesis at the 0.05 significance level.

Fig. 3 shows the proportion of rejected tests for increasing population size and Morgans length threshold with sample size fixed at 5000 and 10,000 diploid individuals. The trend is that the proportion of rejected tests decreases with the increasing population size and Morgans length threshold. Figure S4 shows that this trend does not depend on the significance level. These observations align with the condition n=o(Nw) in Theorems 3.1 and 3.3. The setting for which the proportion is closest to 0.05 is $n=10^{4},\;N=10^{6}$, and w=0.04. Interestingly, for the same sample size and Morgans length threshold, we observe more rejected tests for $N=10^{7}$ than for $N=10^{6}$. This observation aligns with the condition $Nw=o\left(n^{2}\right)$ in Theorems 3.1 and 3.3 (there are too few observed IBD segments).

Figure S5 shows the proportion of rejected tests for increasing sample size and Morgans length threshold with population size fixed at 50,000 and 100,000 diploid individuals. The proportion of rejected tests decreases slightly as the sample size increases. This trend may be explained by the fact that sample size n does not affect the marginal correlations of the IBD segments of three or four specific haplotypes, which are functions of the scaled population size Nw (Lemmas A.3, A.4, and A.5). Provided the sample size n is not too large relative to the scaled population size Nw, the total covariance attributed to ${\text{Cov}}_{3}$ and ${\text{Cov}}_{4}$ terms remains small; meanwhile, increasing the sample size means that we observe more detectable IBD segments.

#### 5.1.2. Flexible demographic scenarios

Figure S6 shows the proportion of rejected tests for increasing sample size and Morgans length threshold in the three phases of exponential growth and population bottleneck demographic scenarios. For Morgans length threshold greater than or equal to 0.03, the proportions of rejected tests are less than 0.3 and 0.1 in the three phases of exponential growth and population bottleneck scenarios, respectively. Consistent with our central limit theorems, we observe a decreasing trend as we increase the Morgans length threshold, even though the proportions of rejected tests around 0.3 and 0.1 are not close to the nominal significance level 0.05. Additionally, these proportions are less than their corresponding proportions in the population of 25,000 diploid individuals (Fig. 3).

The conditions on the global extrema of population sizes in Theorem 4.1 are very stringent. The most recent population sizes have the strongest impact on the covariances of IBD segment indicators. One interpretation of the results in Figure S6 is that the detectable IBD rate around a locus may behave like a normal distribution in demographic scenarios with large recent population sizes, regardless of the not-so-recent population sizes.

#### 5.1.3. Difference of identity-by-descent rates in two samples

We compute the difference in detectable IBD rates around a locus by splitting 5000 diploid individuals into two equally sized subsets. Then, under different experimental conditions, we perform 250 Shapiro–Wilk tests based on empirical distributions of 500 simulations of the test statistic.

Figure S7 shows the proportion of rejected tests for the difference in IBD rates when the population size or Morgans length threshold increase. At the 0.05 significance level, and for all scaled population sizes, between 0.05 and 0.15 percent of tests are rejected. At the 0.10 significance level, and for all scaled population sizes, between 0.10 and 0.30 percent of tests are rejected. There is no apparent trend as either population size or Morgans length threshold increases. One explanation is that any potential overdispersion of ${\bar{\tilde{Z}}}^{0}$ and ${\bar{\tilde{Z}}}^{1}$, relative to the standard normal distribution, may be partially balanced out by considering the difference in rates. Another explanation is the limited power to reject the Shapiro–Wilk null hypothesis in the scope of our computationally feasible experiments.

Across all simulation experiments in Sections 5.1.1, 5.1.2, and 5.1.3, we reject the null hypothesis of normality at rates greater than the Type 1 error rate of 0.05, using the sample sizes and population sizes explored here. These magnitudes are already quite large relative to existing sample sizes and inferred effective population sizes. Nevertheless, the trends of increasing sample size and scaled population size suggest the validity of our central limit theorems.

### 5.2. Percentiles of the finite sample distributions

Next, we investigate possible explanations for rejecting the nominal significance levels at elevated rates. We focus on the upper percentiles of the empirical distribution of our test statistics $\bar{\tilde{Z}}$ and ${\bar{\tilde{Z}}}^{1}-{\bar{\tilde{Z}}}^{0}$. For each batch of simulations, we compute a mean, a standard deviation, and the mean plus 3 or 4 standard deviations. We refer to the means plus 3 or 4 standard deviations as upper bounds in the context of standard normal confidence intervals. Then, we calculate the 99.86501th and 99.99683th percentiles of the test statistic from all of the simulated data for each experimental condition. For example, if the experimental condition is $N=10^{6},\;n=5000$, and w=0.02, and we generate 500 empirical distributions from 1000 simulations, each mean plus three standard deviations is calculated from 1000 simulations, and the 99.86501th is determined from 500,000 simulations. (These percentiles correspond to the standard normal quantiles of Φ(3) and Φ(4), where Φ is the cumulative distribution function of the standard normal random variable.) We multiply the reciprocal of these 99.86501th and 99.99683th percentiles by their corresponding estimated upper bounds (means plus 3 or 4 standard deviations), which we refer to as the relative upper bounds.

#### 5.2.1. The identity-by-descent rate in one sample

Browning and Browning (2020), Temple et al. (2024), and Temple (2024) conduct hypothesis tests to evaluate if the detectable IBD rate $\bar{\tilde{Z}}$ around any specific locus exceeds a genome-wide mean IBD rate. When our central limit theorems hold, we can interpret their hypothesis test as a one-sample one-sided z test. Our estimated upper bounds, which are the mean plus some standard deviations, are intended to mimic their hypothesis tests (Browning and Browning, 2020; Temple et al., 2024).

**Fig. 4.**
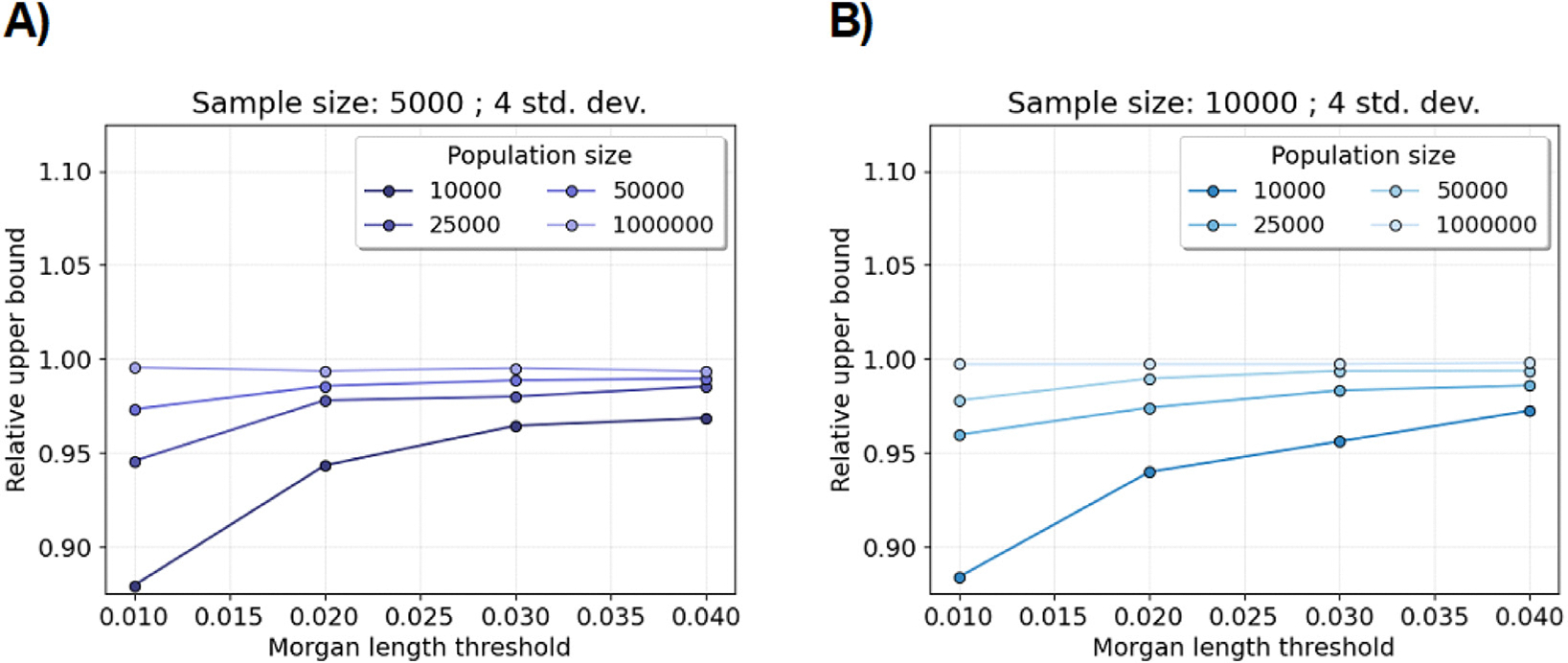
Relative upper bound for excess IBD scan. Line plots show the average mean plus four standard deviations divided by the 99.99683 percentile over two million simulations (y-axis). (The standard normal cumulative distribution function of four is 0.9999683.) Each average relative upper bound is computed over 1000 tests. Each test is based on 2000 simulations of the number of identity-by-descent lengths longer than a specified Morgans length threshold (x-axis). (A) The sample size consists of 5000 diploid individuals. (B) The sample size consists of 10,000 diploid individuals. The legends assign colors to different constant population sizes.

Fig. 4 and S8 show the average relative upper bounds by increasing population size and Morgans length threshold. The average estimated upper bounds are less than the simulated percentile threshold for all sample sizes, population sizes, Morgans length thresholds, and quantiles considered. The average estimated upper bound is proportionally closer to the percentile threshold as population size and Morgans length threshold increase, which is a result consistent with Section 5.1.1 and our central limit theorems.

Figure S6 shows that the average estimated upper bound is also less than the simulated percentile threshold for all sample sizes and Morgans length thresholds in the complex demographic scenarios. The average estimated upper bound is proportionally closer to the percentile threshold for the population bottleneck scenario compared to the three phases of exponential growth scenario, which is the complex demographic scenario with larger recent population sizes (Figure S3).

These experiments suggest that one reason why we reject the Shapiro–Wilk null hypothesis at elevated rates is that the test statistic’s upper tail probability is heavier than that of the standard normal distribution.

#### 5.2.2. Difference of identity-by-descent rates in two samples

Analogous to the excess IBD rate test, the difference in IBD rates ${\bar{\tilde{Z}}}^{1}-{\bar{\tilde{Z}}}^{0}$ may be used as a hypothesis test for equality of means between two labeled subgroups. We perform the same experiment, except that we use the difference in IBD rates as our test statistic.

Figure S9 shows the average relative upper bounds by increasing population size and Morgans length threshold. We see no trend between the average relative upper bounds and sample size, population size, and Morgans length threshold, respectively. Compared to our observation in the one-sample experiment, the upper tail probability of the test statistic is not noticeably different from that of the standard normal distribution. These empirical observations are consistent with our Type 1 error experiment in Section 5.1.3.

### 5.3. Identity-by-descent graphs around a locus

Clusters of detectable IBD haplotypes overlapping a focal point indicate nonnegligible covariance between segments. These cluster covariances could thus explain the observed non-normality in finite samples. We form detectable IBD graphs about a locus by drawing an edge between haplotypes if they share a detectable IBD segment overlapping a focal point. We define detectable IBD clusters as the connected components in the detectable IBD graph.

We use the Erdős–Rényi graph as a baseline to study correlations in the IBD graphs. The Erdős–Rényi graph is a simple network model in which independent edges between nodes occur with a uniform success probability (Erdős and Rényi, 1959). We denote a sparse Erdős–Rényi network as one in which the success probability converges to 0. This contrast analyzes the evolution of independent edges (the Erdős–Rényi graph) versus weakly correlated edges of a specific nature (the IBD graph around a focal point).

For sparse Erdős–Rényi graphs, there are theoretical properties associated with the graph features that we consider in our simulation study. When the success probability is small, the number of trees of order m weakly converges to a Gaussian distribution in large networks (Erdős and Rényi, 1960), and trees of order $m_{1}$ have faster convergence than trees of order $m_{2}$ when $m_{1}<m_{2}$. Another asymptotic property of sparse Erdős–Rényi graphs is that almost all nodes are in trees of small order or a single “giant” component (Erdős and Rényi, 1960). We set the uniform success probability in simulated Erdős–Rényi graphs to be the approximate probability of an IBD segment longer than the Morgans length threshold (Equation 6 in Palamara et al. (2012) for flexible demographic scenarios and Eq. (5) for constant population sizes). Note that the probability of a detectable IBD segment in Eq. (5) goes to 0 as Nw→∞.

Inspired by the above asymptotic behavior of sparse Erdős–Rényi graphs (Erdős and Rényi, 1960), we analyze five features of graphs. The number of edges is equivalent to the number of IBD segments longer than the length threshold. A tree of order m is a connected component that has m nodes and m-1 edges. An order m complete connected component has m nodes and m2 edges between every pair of nodes. While there is no direct connection between the IBD and Erdős–Rényi graphs, we are interested in these features to see if there is empirical evidence of asymptotic behaviors similar to those discussed in Erdős and Rényi (1960).

We count the number of trees of order 2 and 3, the number of complete connected components of order 3 or more, and the number of nodes in the largest connected component. We calculate the average, variance, minimum, and maximum for each feature over replicate simulations. We also conduct Shapiro–Wilk tests by splitting the simulated data as described in Section 5.1.1.

Note that the number of trees of order m is not the same as the m-way IBD rate around a locus. For example, in a complete connected component of four nodes, there are 43 counts of 3-way detectable IBD. As a result, Theorem 4.2 does not apply to the following experiments on tree orders. However, we might expect to see some approximately normally distributed data if most components of degree m are trees.

#### 5.3.1. Comparing to sparse Erdős–Rényi graphs

**Fig. 5.**
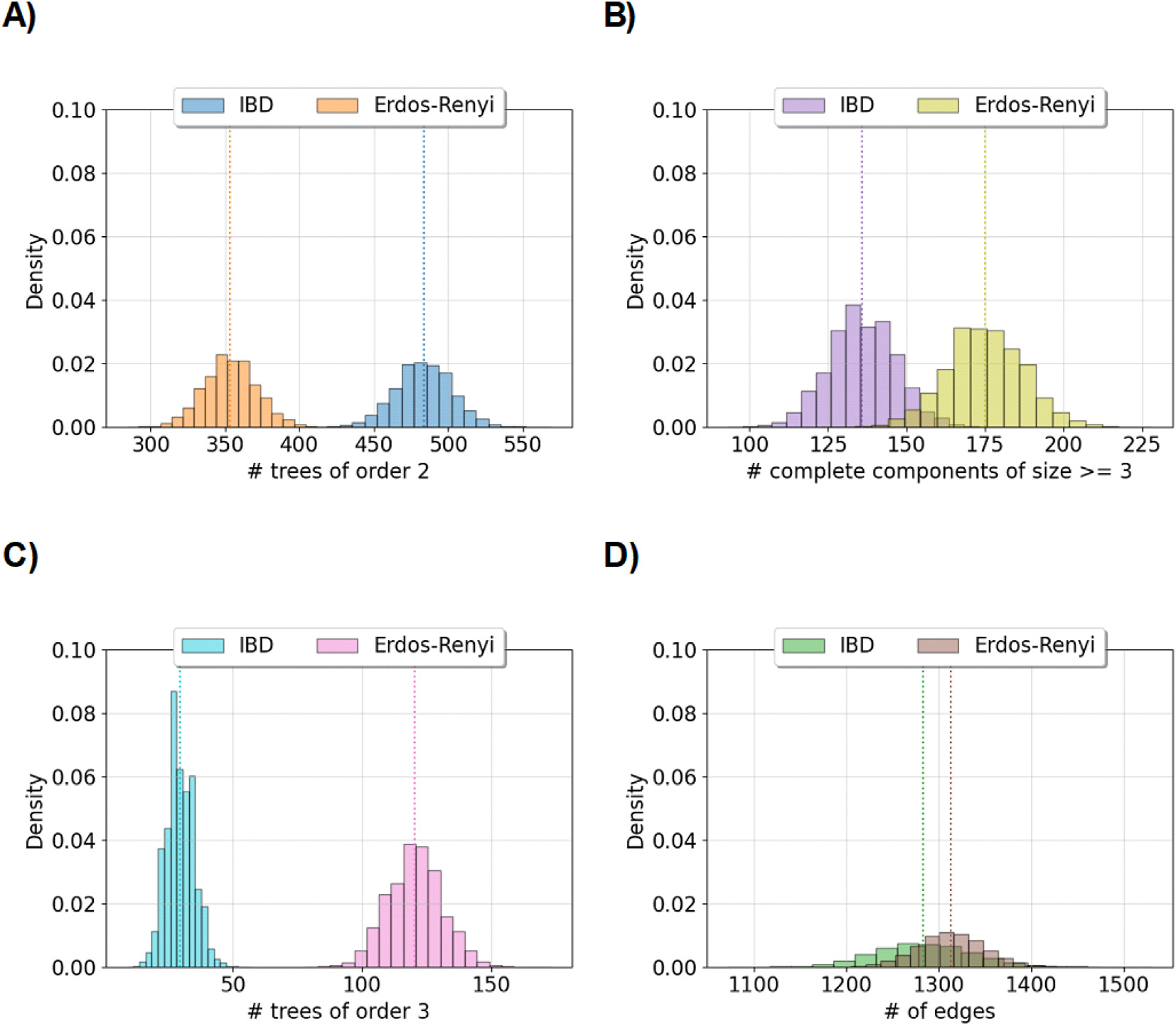
Comparing features between IBD and Erdős–Rényi graphs. Histograms compare the density of graph features between IBD and Erdős–Rényi graphs. Each histogram summarizes the results of 125,000 simulations. (A) and (C) show the number of trees of order 2 and 3, respectively. (B) shows the number of complete components with more than three nodes. (D) shows the total number of edges. The legends assign colors to the IBD and Erdős–Rényi graphs. IBD graphs are simulated using a demography of 100,000 diploid individuals and a 0.03 Morgans length threshold. Erdős–Rényi graphs are simulated using the same success probability as in the IBD graph. The sample size consists of 2000 diploid individuals. Vertical lines show the means.

Fig. 5 shows that some empirical distributions of graph features resemble normal distributions in a sample size of 5000 diploid individuals from a population of 100,000 diploid individuals. Table 2 compares our summary statistics between these simulated detectable IBD and sparse Erdős–Rényi graphs. The variance and maximum number of edges are larger for detectable IBD graphs compared to sparse Erdős–Rényi graphs, which is a direct consequence of the nonzero covariance of IBD edges. (In Fig. 5, the mean IBD rates are different between the number of edges in the IBD graph versus the Erdős–Rényi graph. Note that the expected number of edges should be the same, if not for some approximations (Palamara et al., 2012; Temple et al., 2025).) The proportions of rejected Shapiro–Wilk tests for numbers of trees of order 2 and connected components of degree 3 or more are close to 0.05 for both detectable IBD and sparse Erdős–Rényi graphs. While we observe that some limiting distributional behaviors of small-degree connected components in detectable IBD graphs match those in sparse Erdős–Rényi graphs, these observations go beyond the theory we have presented.

#### 5.3.2. Flexible demographic scenarios

Figure S10 shows that the apparent normality of some graph features extends to the three phases of exponential growth and population bottleneck demographic scenarios. Table S1 reports that the proportions of rejected hypothesis tests for numbers of trees of order 2 are close to 0.05 for both demographic scenarios. We also cannot reject normality for the number of trees of order 3 and the number of connected components of degree 3 or more in some simulations of the three phases of exponential growth scenario. These results indicate that the limiting distributional behaviors of some graph features in detectable IBD graphs around a locus may be similar for large constant populations and demographic scenarios with large recent population sizes.

#### 5.3.3. The impact of strong positive selection

Strong directional selection increases the detectable IBD rate around a locus (Temple et al., 2024) and the probability of IBD alleles (Albrechtsen et al., 2010), but less is known about how this phenomenon alters the feature distributions of detectable IBD graphs. In a hard selective sweep, a single allele increases in frequency at a rate of change that depends on a selection coefficient (Crow and Kimura, 1970; Fisher, 1923; Haldane, 1924; Wright, 1931). The selection coefficient parameterizes the advantage that the sweeping allele has relative to alternative alleles, inasmuch as the gradient of the allele frequency trajectory is larger when the selection coefficient is larger.

We conduct more simulations of detectable IBD graphs for selection coefficients between 0.01 and 0.04 and the three phases of exponential growth and population bottleneck scenarios. Tables S2 and S3 demonstrate multiple trends as the selection coefficient increases. The apparent normality of the number of trees of order 2 does not noticeably change as we change the selection coefficient. Compared to our simulations with no selection, we reject normality less often for the number of trees of order 3 and the number of complete components of order 3 or more. It may be that the distributional behaviors of these small-degree connected components become more apparent under the selection models with more detectable IBD segments. The primary effect of strong positive selection appears to be the expansion of the largest detectable IBD cluster, which includes haplotypes carrying a beneficial allele. This idea is a major motivation for the suite of methods developed in Temple et al. (2024).

## 6. Discussion

In this article, we leverage ideas from coalescent theory and haplotype sharing to develop statistical theory and motivate methodology in IBD-based inference. Most notably, we prove a central limit theorem for the detectable IBD rate around a locus whose regularity conditions have intuitive interpretations in population genetics. The sample size squared must be large enough such that there are many IBD segments long enough to be accurately detected by existing methods (Browning and Browning, 2020; Freyman et al., 2021; Shemirani et al., 2021; Zhou et al., 2020a). The population size must be large enough that there are few to no large IBD clusters about a locus.

The conceptual framework for these conditions involves envisioning a coalescent tree with long internal branches, but numerous coalescent events occur near the leaves. The internal branches are long due to the large population size, and there are numerous coalescent events near the leaves, resulting from the large sample size. The large Morgans threshold further decreases the probability of a detectable IBD segment and the correlations between IBD segment indicators.

The techniques we use might be helpful in other studies involving coalescent *and* recombination processes. For instance, to generalize our main central limit theorem, we take a formulaic approach. First, we derive covariances for a finite set of classes. Second, we count the number of covariance terms of each class that occur in the total covariance of the sample mean. Third, we determine a “little-o” condition such that the sum of covariances of one specific class is asymptotically equivalent to the sum of covariances of all the other classes. We use a particular central limit theorem for dependent data (Chandrasekhar and Jackson, 2025; Chandrasekhar et al., 2023), which is derived using Stein’s moment-based method—a more general technique to demonstrate weak convergences to Gaussian or non-Gaussian random variables (Lange, 2010; Ross, 2011; Stein, 1972, 1986). Future work could use our approach to try to prove central limit theorems for cohort-averaged IBD sharing (Carmi et al., 2013).

We employ simulation to evaluate the assumptions and validity of our central limit theorem. Consistent with our conditions, we reject the null hypothesis of normality less often as sample size and scaled population size increase. In practice, we find that non-normality is typical in finite samples. Carmi et al. (2013) also observe inflated non-Gaussian tails in the empirical distribution of cohort-averaged IBD sharing across the entire genome (Figure 6 in Carmi et al. (2013)). For our work, deviation from normality may be unavoidable in real data because of slow convergence rates (Figure S1). In Section 5.3, we indicate that nonnegligible covariance of the IBD rate may come from the accumulation of IBD clusters. Based on the tail behavior of simulated distributions, we expect that a one-sample z test for excess IBD rates may inflate the number of false positives.

Our regularity conditions concern a balance between sample size and scaled population size that is unlikely to hold in practical settings. In our experiments, we observe neither a trend between sample size and the proportion of rejected tests nor between sample size and the relative upper tail probability. We advocate that the collected sample size should always be as large as is feasible and that the smallest Morgans length threshold for which IBD segment detection is accurate should be chosen. For high-quality genetic data on humans, we recommend using a segment detection threshold of 0.02 or 0.03 Morgans.

Our theoretical results and simulation studies support ongoing methodological developments based on IBD segments. Existing genome-wide scans for excess IBD rates (Browning and Browning, 2020; Temple et al., 2024) or differences in IBD rates between groups (Browning and Thompson, 2012) lack formal or exact hypothesis testing frameworks. Motivated in part by this work, Temple (2024) controls the family-wise error rate (FWER) in their selection scan by modeling the IBD rate process as an Ornstein–Uhlenbeck process, thereby assuming that the IBD rate is normally distributed at any given spatial position. Consistent with this work, they demonstrate anti-conservative control of the false discovery rate (FWER). Combining an FWER control technique (Feingold et al., 1993; Siegmund and Yakir, 2007) with our multivariate central limit theorem, we indicate that a modification of the Temple (2024) method may apply to a test for equality of detectable IBD rates in case-control studies. In these examples and others (Conneely and Boehnke, 2007; Grinde et al., 2019; Liu et al., 2019) from statistical and population genetics, assuming reasonable asymptotic models is often vital when adjusting for many correlated tests.

## Supplementary Material

1

## Figures and Tables

**Table 1:** Glossary of mathematical terms. Precise definitions are provided in the main text.

Term	Definition

n	Sample size
N	Constant population size
N(t)	Population size at time t
a, b, c, d	Indices for sample haplotypes
$L_{a}$, $R_{a}$	Sample a’s endpoints to the left and right of a focal point
$L_{a,b,}$, $R_{a,b}$	Left and right endpoints that are shared by a and b
$W_{a,b}$	IBD segment around a focal point that is shared by a and b
w	Segment length threshold
$X_{a,b}$	Indicator that $R_{a,b}$ exceeds w
$Y_{a,b}$	Indicator that $W_{a,b}$ exceeds w
${\bar{Z}}_{a,b}$ and ${\bar{\tilde{Z}}}_{a,b}$	Standardized sample means of $\left\{X_{a,b}\right\}$ and $\left\{Y_{a,b}\right\}$, respectively
$Z_{-a,b}$ and ${\tilde{Z}}_{-a,b}$	Sum over all indicators except $X_{a,b}$ and $Y_{a,b}$, respectively
Ω	Sum of the variances of all IBD segment indicators
$E_{m}$	Expectation integrated over m haplotypes
${Cov}_{m}$	Covariance integrated over m haplotypes
Superscript l	Denotes the label of a sample set
Subscript nm	Denotes summation or average over nm indicators
Subscript N	Denotes the constant population size

**Table 2:** Summary statistics of IBD and Erdős–Rényi graphs. Network structures of interest are the number of edges (Edges), the degree of the largest components (Largest), the number of trees of order 2 and 3 (Tree-2 and Tree-3), and the number of complete components of degree 3 or more (Complete). Summary statistics are aggregated over 125,000 simulations. Shapiro–Wilk tests at the significance level 0.05 are performed with 500 replicates for 250 simulations, and the proportion of rejected null hypotheses is reported as S.W.t. The population size is 100,000 diploid individuals. The sample size consists of 2000 diploid individuals. The Morgans length threshold is 0.03.

Type	Structure	Avg	Var	Min	Max	S.W.t.

IBD	Edges	1283.42	2690.85	1072.00	1530.00	0.14
	Largest	8.09	1.81	5.00	22.00	1.00
	Tree-2	483.62	346.48	402.00	569.00	0.05
	Tree-3	29.40	28.38	9.00	57.00	0.81
	Complete	135.89	112.45	93.00	187.00	0.18

Erdős–Rényi	Edges	1312.68	1313.06	1158.00	1475.00	0.07
	Largest	27.02	74.07	11.00	137.00	1.00
	Tree-2	353.31	310.32	284.00	434.00	0.08
	Tree-3	120.31	109.73	78.00	173.00	0.14
	Complete	174.94	146.10	123.00	228.00	0.13

## Data Availability

We use the Python package https://github.com/sdtemple/isweep for all simulation studies. This software is freely available under the open-source CC0 1.0 Universal License.

## Appendix A. Supplementary data

Supplementary material related to this article can be found online at https://doi.org/10.1016/j.tpb.2025.06.003.
